# Behavioral changes and dendritic remodeling of hippocampal neurons in adolescent alcohol-treated rats

**DOI:** 10.3389/adar.2023.11158

**Published:** 2023-07-24

**Authors:** Ratna Sircar

**Affiliations:** ^1^ Department of Psychology, The City College of New York, City University of New York, New York, NY, United States; ^2^ Department of Psychiatry and Behavioral Sciences, Albert Einstein College of Medicine, Bronx, NY, United States

**Keywords:** teenage drinking, alcohol blackout, contextual fear conditioning, dendritic branching, cued fear memory

## Abstract

**Objective:** Earlier, we and others have reported that alcohol exposure in adolescent rat impaired performance of a spatial memory task in the Morris water maze. The goal of the present study was to investigate the effects of acute adolescent alcohol treatment on the hippocampus-dependent (contextual fear conditioning) and hippocampus-independent (cued fear) memories. The study also looked at the structural changes in anterior CA1 hippocampal neurons in adolescent alcohol-treated rats.

**Methods:** Adolescent female rats were administered with a single dose of alcohol (1.0, 1.5, or 2.0 g/kg) or vehicle either before training (pre-training) or after training (pre-testing). Experimental and control rats were trained in the fear conditioning paradigm, and 24 h later tested for both contextual fear conditioning as well as cued fear memory. Separate groups of rats were treated with either alcohol (2 g/kg) or vehicle and sacrificed 24 h later. Their brains were harvested and processed for rapid Golgi staining. Randomly selected CA1 pyramidal neurons were analyzed for dendritic branching and dendritic spine density.

**Results:** Pre-training alcohol dose-dependently attenuated acquisition of hippocampus-dependent contextual fear conditioning but had no effect on the acquisition of amygdala-associated cued fear. When administered following training (pre-testing), alcohol did not alter either contextual conditioning or cued fear memory. Golgi stained CA1 pyramidal neurons in alcohol treated female rats had reduced basilar tree branching and less complex dendritic arborization.

**Conclusion:** Alcohol specifically impaired hippocampal learning in adolescent rats but not amygdala-associated cued fear memory. Compared to vehicle-treated rats, CA1 hippocampal pyramidal neurons in alcohol-treated rats had less complex dendritic morphology. Together, these data suggest that adolescent alcohol exposure produces changes in the neuronal organization of the hippocampus, and these changes may be related to impairments in hippocampus-dependent memory formation.

## Introduction

According to the NIAAA, alcohol is the number one substance misused by adolescents, far outnumbering those using all illicit drugs combined. In 2019, 18.7% of youth between 12 and 20 years of age reported having consumed alcohol in the past 30 days, with the median age for initiation of alcohol drinking being around 15 years [1, 2]. Alcohol exposure in adolescents causes significant deficits in executive functioning, visuo-spatial skills, attention, and verbal and nonverbal memory [3, 4]. Alcohol-induced partial or complete blocking of memory formation, known as alcohol blackout, is quite prevalent among adolescents [5, 6]. The highest rate of such memory loss (32%) is seen in 16- and 17-year-olds [7].

Alcohol has also been reported to cause memory deficits in adolescent animals. Studies from our laboratory and others have reported that in adolescent rats alcohol impairs spatial memory task performance in the Morris water maze, a hippocampus-dependent behavioral test [8, 9]. Another hippocampus related behavior modulated by alcohol in adolescent rats is contextual fear conditioning. Acute administration of alcohol in adolescent rats has been reported to impair contextual fear conditioning [10]. When tested as adults, repeated alcohol exposure in adolescent rats caused deficits in context fear conditioning but not in cued fear memory [11]. Gender differences have been reported in alcohol-induced memory functions both in humans and animals, with females being more susceptible to alcohol’s amnesic effects than males [10, 12–14].

Alcohol affects memory in an age-dependent manner. Several studies have reported that compared to adult animals, adolescent animals are more sensitive to the amnesic effects of alcohol. Compared to adult rats, adolescent rats show greater alcohol-induced memory impairments in the Morris water maze task [8, 9]. In an appetitive odor discrimination task, alcohol has been shown to produce greater impairment in adolescent animals than adult animals, [15]. Hippocampal slices from adolescent rats show higher sensitivity to alcohol-induced inhibition of long-term potentiation than slices from adult rats [16]. Although the disruptive effects of alcohol on memory in adolescent animals is well documented, research into alcohol’s hippocampus-associated cognitive impairments in adult animals is less consistent [17]. Moderate doses of acute ethanol (1.5–2.0 g/kg) in rats have been shown to impair contextual memory [18] but it did not seem to impair spatial cognition directly but appeared to increase general response perseveration, reduce behavioral flexibility, and impair performance in reversal tasks via mechanisms other than hippocampal function [19].

Hippocampus and related structures are intimately involved in cognition, particularly learning and memory [17], and is important in spatial memory and contextual conditioning [20, 21]. Pharmacological and structural lesioning of the hippocampus have been shown to impair hippocampus-related learning [22, 23]. Following hippocampal lesion, animals trained in the fear-conditioning paradigm demonstrated impaired memory when tested to the conditioned context, but not to cue testing [24]. Whereas, hippocampus is critical for learning the context [24], amygdala is important for cued learning [25, 26].

Since the hippocampus undergoes progressive development throughout adolescence, it is thought to be especially susceptible to alcohol’s impairing effects on neurocognitive functioning, including alcohol blackout [27, 28]. Structural changes in the hippocampal brain region have been reported in young alcohol misusers who show memory impairment [29]. Adolescents with alcohol use disorder have reduced hippocampal grey matter volumes as well as alterations in synapse structure and function that are of fundamental importance in learning and memory [30, 31]. The present study was designed to study brain region-specific alcohol-induced learning effects, and to investigate adolescent alcohol-induced morphological alterations in hippocampal CA1 pyramidal cells.

## Materials and methods

Subjects: Ninety-six adolescent (PD25 at the beginning of experiment) Sprague Dawley female rats (Taconic, Germantown, NY, United States) were used in the study. Rats were housed in the temperature- and humidity-controlled institutional animal facility and maintained on a 12:12 h light/dark cycle (lights on at 7:00 AM). Rats had *ad libitum* access to food and water. All experimental protocols were approved by the Institution’s Animal Care and Use Committee, and in compliance with the Guide for the Care and Use of Laboratory Animals (National Research Council, 2011). Female rats were used since it has been reported that male rats are relatively insensitive to alcohol’s amnesic effect [10].

Alcohol administration: Rats received a single intraperitoneal injection (1.0, 1.5, or 2 g/kg) of alcohol (25% v/v) or vehicle (saline). Doses of alcohol selected were based upon our earlier studies [8, 10, 13, 32]. Each rat received a single injection of alcohol (one of three doses) or vehicle either 30 min before behavioral training (pre-training group) or 30 min before behavioral testing (pre-testing group).

Apparatus: Fear conditioning was conducted in a Plexiglas rodent conditioning chamber (28 cm × 21 cm × 21 cm) with a metal grid floor, lit with a single house light and enclosed within a sound-attenuating cubicle (Med Associates, St. Albans, VT). The floor grid was connected to a shock generator and scrambler (Med Associates, St. Albans, VT) for the delivery of an electric foot shock. Foot shock was used as an unconditioned stimulus (US). The chamber was also equipped with an electronic alarm with a speaker (Mallory Sonalert, Indianapolis, IN) for the delivery of a tone that served as the conditioned stimulus (CS). The same conditioning chamber as used for training was used for testing contextual fear memory. For testing of cued fear memory, the behavioral chamber was modified with the introduction of a removable rectangular partition placed diagonally through the middle of the chamber, one wall was lined with a novel design, and the floor as covered with a flat piece of smooth Plexiglas.

Fear conditioning: Each rat was placed in the conditioning chamber and presented with a continuous tone (2.9 kHz, 80 dB) for 30 s, at the end of which an electric shock (1 mA) was delivered through the floor grid for 2 s that co-terminated with the tone. Each rat was subjected to two tone (CS)-shock (US) pairings with an interval of 60 s between the sessions. Approximately 24 h after training, all animals were tested for contextual fear conditioning and auditory cued fear. Each rat was put through both behavioral tasks. The sequence of testing was counterbalanced so that the order of testing did not affect behavioral outcomes.

Experimental design: On the training day, each animal was brought from the home cage to the behavioral suite and allowed to rest for 30 min. Rats were injected intraperitoneally with one of three doses of alcohol (1.0, 1.5, or 2.0 g/kg) or vehicle 30 min before behavioral training (pre-training group). Control rats received equivalent volumes of vehicle injection. Rats were put through two US-CS pairings that were 60 s apart. Following the second training session, animals were returned to their home cages. Twenty-four hours later, rats were brought back from their home cages to the behavioral suite and allowed to rest for 30 min. For the pre-testing group, each rat received one of three doses of alcohol (1.0, 1.5, or 2.0 g/kg) or vehicle 30 min before behavioral testing. All rats were tested for both contextual fear conditioning and cued fear memory. For the test of contextual fear conditioning, rats were placed in the same conditioning chamber as that used for training and observed for freezing behavior. No foot shock or sound stimulus was given. For testing of cued fear, the animal was placed in the modified chamber (as described before) and subjected to sound stimulus. Each rat was given both behavioral tests and the order was randomized to avoid sequence effect.

Freezing behavior was observed for 3 min. No sound or foot shock was given for contextual fear testing. For cued fear test, sound stimulus was given. In both behavioral tests, each animal’s behavior was manually scored every 10 s on a three-point scale (0: moving; 1: exhibiting head movements only; 2: not moving except for respiration) to determine whether freezing occurred within each 10-s bin. The scores were summed and freezing scores were obtained [32].

Data analysis: SPSS for Windows (IBM, New York) was used for statistical comparisons. Each experimental group had 9–12 rats. Behavioral data was analyzed using one-way analysis of variance (ANOVA) followed by Tukey’s HSD *post hoc* test. The level of significance was set at *p* < 0.05.

Golgi staining: Separate groups of rats were injected intraperitoneally with a single dose of alcohol (2 g/kg alcohol; *n* = 6) or vehicle (*n* = 6) and sacrificed 24 h later. Brains were isolated and fixed in 10% neutral buffered formalin. Tissue blocks were cut, fixed, and stained using the rapid Golgi method (Neurostructural Research Lab, Tampa, FL). Whole brains were subjected to silver impregnation for 2 weeks and cryoprotected for 48 h. Fixed blocks were placed in a solution containing osmium tetroxide and potassium dichromate, immersed in silver nitrate, dehydrated, and infiltrated with nitrocellulose. Nitrocellulose-hardened tissue sections were cut at 120 μ thickness, cleared in alpha terpineol, rinsed in xylene, and coverslipped on coded slides under Permount^®^.

Analysis of hippocampal dendritic morphology: CA1 pyramidal neurons were randomly selected from coded slides for analyses of dendritic spines and dendritic branching. Dendritic arbors were analyzed using Sholl analysis and branch point analysis. For Sholl analysis, concentric circles or “shells” were superimposed over the camera lucida drawings. The radius of the first shell was 10 μm from the cell soma and subsequent shells increased by 10 μm increments out to 200 μm away from the soma. Interactions were measured by the number of times a dendrite intersected with a shell. Branch point analysis measured the number of dendrite branch bifurcations. A first-order branch was a branch emanating directly from the soma. The point where this branch split into two was considered as a first-order branch point, and the resultant branches were second-order branches. This pattern was repeated until the branches reached the endpoint; more branch points indicated increased dendritic branching complexity [33].

Data analysis: For statistical comparison, anatomical data was analyzed using Wilcoxon signed rank test. The level of significance was set at *p* < 0.05.

## Results

**FIGURE 1:**
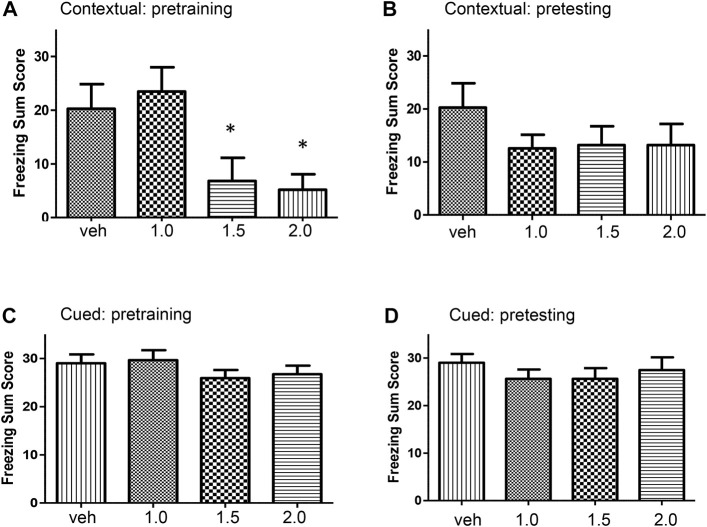
**(A)** Dose-dependent effects of alcohol on the acquisition of contextual fear conditioning. Rats were injected intraperitoneally with one of three doses of alcohol (1.0, 1.5, or 2.0 g/kg) or equivalent volumes of vehicle. There was a significant treatment effect on freezing summed score (F [3, 41] = 4.91, *p* < 0.005). Post hoc comparisons showed that rats treated with 1.5 g/kg (*p* = 0.034) and 2 g/kg (*p* = 0.018) alcohol froze significantly less than the control group. **(B)** Effects of pre-testing alcohol administration, i.e., following training, on contextual fear conditioning. Rats were trained for fear conditioning and twenty-four hours later they were injected with one of three doses of alcohol (1.0, 1.5, or 2.0 g/kg) or equivalent volume of vehicle. Pre-testing (post-training) alcohol did not alter freezing summed score (F [3, 41] = 0.96, *p* = 0.42). **(C)** Effects of alcohol administration on the acquisition of cued fear memory. Rats were injected with one of three doses of alcohol (1.0, 1.5, or 2.0 g/kg) or equivalent volume of vehicle and trained for fear conditioning. Pre-training alcohol did not alter cued fear learning (F [3, 41] = 0.91, *p* = 0.44). **(D)** Effects of pre-testing alcohol administration on cued fear. Rats were trained for fear conditioning. Twenty-four hours later rats were injected with one of three doses of alcohol (1.0, 1.5, or 2.0 g/kg, intraperitoneal) or equivalent volume of vehicle and tested for cued fear memory in a modified chamber in the presence of sound stimulus. Pre-testing (post-training) alcohol did not affect cued fear memory (F [3, 41] = 0.56, *p* = 0.64). alc, alcohol; veh, vehicle. Values indicate mean ± s.e.m.; *n* = 9–12 rats per group; *p < 0.05.

**Pre-training alcohol on the acquisition of contextual freezing** Adolescent rats were treated with a single dose of alcohol (1.0, 1.5, or 2.0 g/kg) or vehicle 30 min before training in the conditioning chamber and tested 24 h later for contextual freezing. There was a significant effect of acute alcohol on contextual freezing memory (F [3, 41] = 4.91, *p* = 0.005). Alcohol dose-dependently reduced freezing summed score (Figure 1A). Contextual freezing summed scores in alcohol-treated rats (1.5 and 2.0 g/kg) were significantly lower than in control rats (*p* < 0.05).

**Pre-testing alcohol on contextual fear conditioning** Adolescent rats were trained in the conditioning chamber. Twenty-four hours later, groups of rats were injected with a single dose of alcohol (1.0, 1.5, or 2.0 g/kg) or vehicle and 30 min later tested for contextual freezing in the same chamber. There was no significant effect on contextual fear conditioning when alcohol was administered before testing (F [3, 41] = 0.96, *p* = 0.42). Administration of alcohol after training did not affect contextual fear conditioning (Figure 1B).

**Pre-training alcohol on the acquisition of cued fear memory** Adolescent rats were treated with a single alcohol dose (1.0, 1.5, or 2.0 g/kg) or vehicle, 30 min before training in the conditioning chamber and tested 24 h later in the modified conditioning chamber along with sound stimulus. There was no effect of alcohol on learning of cued fear (F [3, 41] = 0.91, *p* = 0.44). Alcohol did not alter acquisition of cued fear memory (Figure 1C).

**Pre-testing alcohol on cued fear memory** Adolescent rats were trained in the conditioning chamber. Twenty-four hours later, groups of rats were treated with a single dose of alcohol (1.0, 1.5, or 2.0 g/kg) or vehicle, and 30 min later tested in the modified conditioning chamber along with sound stimulus. There was no significant effect of pre-testing alcohol on cued fear (F [3, 41] = 0.56, *p* = 0.64). Alcohol did not alter cued fear memory when administered after training (Figure 1D).

**FIGURE 2:**
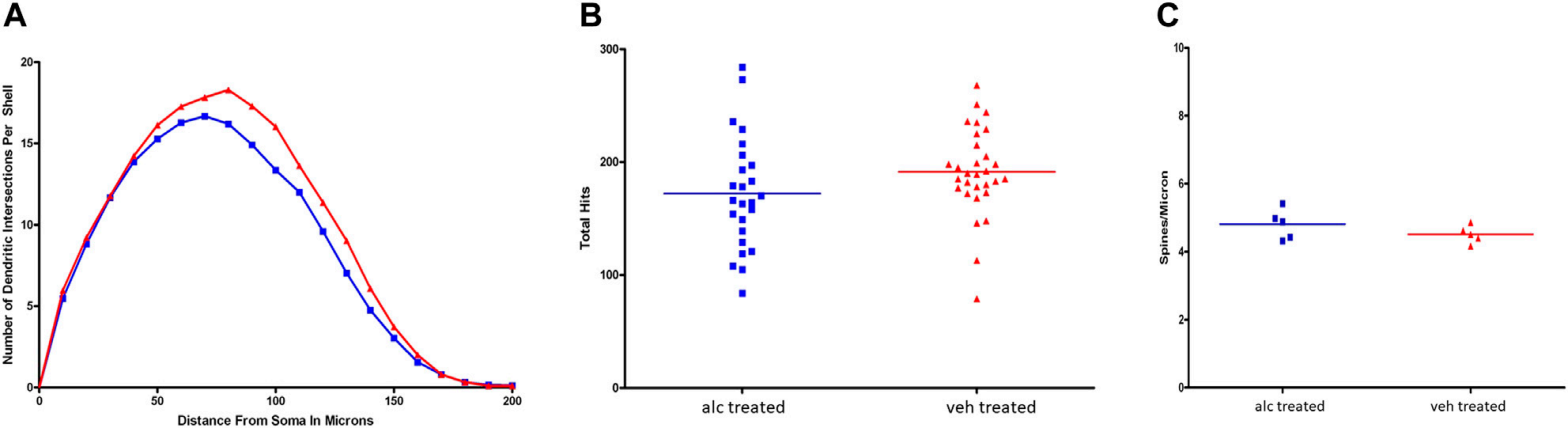
**(A)** Comparison of Sholl profiles of CA1 pyramidal neuronal dendritic branching. Sholl profile of vehicle-treated CA1 neurons (red) was significantly greater (*p* = 0.0003) than profile of alcohol-treated CA1 neurons (blue). **(B)** Branch point analysis. Results of total basilar branch point analyses showed that there was a significant difference between the alcohol (blue) and vehicle (red) profiles, with *p* = 0.0331. Alcohol-treated CA1 pyramidal neurons had fewer basilar branch points than vehicle-treated ones suggesting that the CA1 dendritic arborization was less complex than controls. **(C)** There was no significant difference in apical thin-type spine densities per CA1 pyramidal neuron between vehicle-treated (red) and alcohol-treated (blue) groups.

**Alcohol on dendritic morphology of hippocampal pyramidal neurons** Anterior CA1 pyramidal neurons were randomly selected from coded slides for analyses of dendritic branching and spine density. Results of Sholl analysis and branch point analysis showed significant differences between the experimental and control groups (Figure 2). Comparison of Sholl profiles indicated that the vehicle-treated CA1 pyramidal neuronal profile was significantly greater than alcohol-treated profile, with a *p*-value of 0.0003 (Figure 2A). The results of Wilcoxon test comparing the two dendritic profiles for branch point analysis showed significant differences between the two profiles (*p* = 0.03). Vehicle-treated CA1 pyramidal neurons had significantly more basilar branch points than alcohol-treated CA1 pyramidal neurons, indicating that dendritic arborization of CA1 pyramidal neurons in alcohol-treated rats was less complex than in control animals. Data shown in Figure 2B. The thin apical spine density on CA1 pyramidal neuronal dendrites in alcohol-treated anterior hippocampus did not differ from control spine density (Figure 2C).

## Discussion

In the present study, conditioned fear was used to study subject effects of alcohol on two different forms of learning, the hippocampus-dependent contextual fear conditioning and hippocampus-independent, amygdala-based cued fear [34]. In adolescent rats, acute alcohol when administered before training in the conditioning paradigm resulted in impaired acquisition of contextual fear conditioning. When administered after learning had taken place, alcohol had no significant effect on contextual fear memory. The effect of alcohol on contextual fear conditioning was dose dependent. A dose of 1.0 g/kg had minimal effect on contextual fear conditioning, while doses of 1.5 and 2.0 g/kg significantly disrupted context conditioning. Acute alcohol in adolescent rats did not affect cued fear memory irrespective of when the adolescent rats were given alcohol, whether before training (pre-training) or after training (pre-testing). Acute alcohol (2 g/kg) exposure altered the CA1 pyramidal neuronal dendritic architecture. Compared to CA1 pyramidal neurons in vehicle-treated rats, alcohol-treated CA1 pyramidal neurons had reduced basilar tree branch points and dendritic arborization was less complex than in controls. The present data indicates that in adolescent rats acute alcohol specifically impaired the acquisition of hippocampus-related contextual fear conditioning but not hippocampus-independent, amygdala-associated cued fear memory. These findings support data from earlier studies reporting that alcohol administration in adolescent rats impaired performance in the Morris water maze hidden platform task, a form of spatial memory that is dependent on the hippocampus [8, 9, 13].

Alcohol-induced blackouts are common among adolescents. Blackouts are periods of alcohol-induced anterograde amnesia. Subjects exhibit deficits in learning and encoding and are unable to form new long-term memories while intoxicated but can recall memories formed prior to intoxication [35]. In the present study, adolescent rats showed a reduction in freezing score when alcohol was administered prior to the presentation of tone–shock pairing indicating deficits in learning. When alcohol was administered after tone–shock pairings, there was no effect on freezing scores suggesting that memory once formed was not affected. Thus, alcohol in adolescent rats mimicked human alcohol-induced blackouts. Acute alcohol in adolescent rats impaired learning but not memory once formed. Blackout occurs when the blood alcohol level is around 150 mg/dL or higher [36]. The present study did not record blood alcohol levels. Earlier we have reported that blood alcohol levels following an intraperitoneal injection of 2 g/kg alcohol resulted in blood alcohol levels of 124 and 311 mg/dL at 30 and 60 min post-injection, respectively [13]. The doses of alcohol used in the present study were based upon previous studies from our laboratory and from literature [8, 9, 13, 37].

Following alcohol treatment, adolescent animals trained in a standard fear-conditioning task exhibit impaired memory when tested to the conditioned context, but did not exhibit impaired memory in cue testing. Alcohol effects on hippocampal behavior in adult animals have been less consistent [17], and the effects have been dose-dependent and task-dependent. Pretraining alcohol in adults failed to affect trace conditioning at lower doses (0.5 and 1.0 g/lg) and impaired trace conditioning only at high dose [37]. Some studies have reported that alcohol exposure impairs spatial cognitive memory in adult animals [38] but others did not [8, 9]. In another study low doses of alcohol in adult animals have been shown to facilitate spatial working memory under certain challenging test conditions [39]. In still other studies, alcohol in adult rodents disrupted acquisition of both contextual conditioning as well as cued fear [34, 40].

In the present study the effects of alcohol on memory were examined in female adolescent rats. Earlier, we have reported that female animals were more sensitive to alcohol effects than male rats [10]. In a study where adolescent animals were injected with alcohol prior to training for trace conditioning and tested as adults, showed alcohol-related reduction in context fear. The effect was especially pronounced in females [37].

Hippocampus has been demonstrated to be involved in spatial learning and memory, contextual learning, trace conditioning, and spontaneous alternation [11]. The hippocampus is also critically sensitive to alcohol. Since learning circuitry is still developing throughout adolescence [41, 42], it is highly likely that alcohol exposure affects learning processes by modifying hippocampal architecture. The present data supports this by demonstrating that exposure to alcohol during adolescence produced changes in the neuronal organization in the hippocampus. The major effect of adolescent alcohol exposure was a decrease in basilar dendritic branching of the anterior hippocampal CA1 region. Dendrites form functional contacts with neighboring axons of other neurons. Alcohol exposure during adolescence appears to inhibit the normal developmental trajectory of CA1 pyramidal neuron dendrite complexity, and since these neurons play an integrative role in contextual fear conditioning, adolescent alcohol-induced adaptations in CA1 dendrite complexity may be contributing to the impairment of acquisition of contextual conditioning. One possible mechanism could be that alcohol damages specific afferents to the CA1 cells and thereby cause transneuronal degeneration of portions of the proximal basilar dendrites. This needs to be examined in future studies.

Changes in size, shape, and density of synaptic spines are associated with learning and memory [43]. Dendritic spines are the principal site for excitatory transmission in the brain [44]. In adult rats, alcohol causes dendritic spine loss particularly in the posterior hippocampus, suggesting increased sensitivity of posterior hippocampus to adult alcohol [45]. Thin spines, considered as “learning spines,” are thought to be transient spines and are increased during memory acquisition [46]. In the present study, the number of thin spines per neuron did not change following acute adolescent alcohol administration. Other types of spines, e.g., mushroom and stubby spines not examined here, may be altered by adolescent alcohol. Dendritic morphogenesis is regulated by extrinsic factors such as neurotropic factors, cell adhesion molecules, and activity dependent calcium signaling, as well as by intrinsic factors such as transcription factors and cytoskeletal regulators [47]. Modulation of the cytoskeleton is critical for regulating spine plasticity, and alterations in dendritic actin cytoskeletal dynamics can impair dendritic arborization. Future studies will explore mechanisms underlying altered dendritic morphology of hippocampal pyramidal neurons in alcohol-treated adolescent brain.

## Conclusion

To summarize, in adolescent rat acute alcohol treatment produced impairments in the acquisition of contextual fear conditioning. Alcohol when administered after rats had been trained in the tone-shock pairing paradigm did not alter contextual fear conditioning. Alcohol did not affect cued fear memory when administered either before training or after training. These data suggest the specificity of acute adolescent alcohol exposure on hippocampal learning. Adolescent alcohol exposure modulated the dendritic tree architecture in the anterior hippocampal CA1 pyramidal neurons suggesting that the anterior CA1 hippocampus is an important locus for adolescent alcohol-induced neuroadaptations, and this may be associated with adolescent alcohol blackouts.

## Data Availability

The raw data supporting the conclusion of this article will be made available by the authors, without undue reservation.
